# Developing a Machine Learning Model to Predict Severe Chronic Obstructive Pulmonary Disease Exacerbations: Retrospective Cohort Study

**DOI:** 10.2196/28953

**Published:** 2022-01-06

**Authors:** Siyang Zeng, Mehrdad Arjomandi, Yao Tong, Zachary C Liao, Gang Luo

**Affiliations:** 1 Department of Biomedical Informatics and Medical Education University of Washington Seattle, WA United States; 2 Medical Service San Francisco Veterans Affairs Medical Center San Francisco, CA United States; 3 Department of Medicine University of California San Francisco, CA United States

**Keywords:** chronic obstructive pulmonary disease, machine learning, forecasting, symptom exacerbation, patient care management

## Abstract

**Background:**

Chronic obstructive pulmonary disease (COPD) poses a large burden on health care. Severe COPD exacerbations require emergency department visits or inpatient stays, often cause an irreversible decline in lung function and health status, and account for 90.3% of the total medical cost related to COPD. Many severe COPD exacerbations are deemed preventable with appropriate outpatient care. Current models for predicting severe COPD exacerbations lack accuracy, making it difficult to effectively target patients at high risk for preventive care management to reduce severe COPD exacerbations and improve outcomes.

**Objective:**

The aim of this study is to develop a more accurate model to predict severe COPD exacerbations.

**Methods:**

We examined all patients with COPD who visited the University of Washington Medicine facilities between 2011 and 2019 and identified 278 candidate features. By performing secondary analysis on 43,576 University of Washington Medicine data instances from 2011 to 2019, we created a machine learning model to predict severe COPD exacerbations in the next year for patients with COPD.

**Results:**

The final model had an area under the receiver operating characteristic curve of 0.866. When using the top 9.99% (752/7529) of the patients with the largest predicted risk to set the cutoff threshold for binary classification, the model gained an accuracy of 90.33% (6801/7529), a sensitivity of 56.6% (103/182), and a specificity of 91.17% (6698/7347).

**Conclusions:**

Our model provided a more accurate prediction of severe COPD exacerbations in the next year compared with prior published models. After further improvement of its performance measures (eg, by adding features extracted from clinical notes), our model could be used in a decision support tool to guide the identification of patients with COPD and at high risk for care management to improve outcomes.

**International Registered Report Identifier (IRRID):**

RR2-10.2196/13783

## Introduction

### Background

In the United States, chronic obstructive pulmonary disease (COPD) affects 6.5% of adults [1] and is the fourth leading cause of death, excluding COVID-19 [2]. Each year, COPD causes 1.5 million emergency department (ED) visits, 0.7 million inpatient stays, and US $32.1 billion in total medical cost [1]. Severe COPD exacerbations are those that require ED visits or inpatient stays [3], account for 90.3% of the total medical cost related to COPD [4], and often cause irreversible decline in lung function and health status [5-10]. Many severe COPD exacerbations (eg, 47% of the inpatient stays for COPD) are deemed preventable with appropriate outpatient care [3,11] because COPD is an ambulatory care–sensitive condition [12]. A commonly used method to reduce severe COPD exacerbations is to place patients at high risk in a care management program for preventive care [13-15]. Patients at high risk can be identified prospectively using a predictive model [16]. Once a patient enters the care management program, a care manager will periodically contact the patient for health status assessment and to help coordinate health and related services. This method is adopted by many health plans, such as those in 9 of 12 metropolitan communities [13], and many health care systems. Successful care management can reduce up to 27% of the ED visits [14] and 40% of the inpatient stays [15] in patients with COPD.

However, because of limitations of resources and service capacity, only ≤3% of patients could enter a care management program [17]. Its effectiveness is upper bounded by these patients’ risk levels, which are determined by how accurate the used predictive model is. Neither the stage of COPD nor having prior severe COPD exacerbations alone can predict a patient’s risk level for future severe COPD exacerbations well [18,19]. Previously, researchers had built several models to predict severe COPD exacerbations in patients with COPD [20-53]. These models are inaccurate and suboptimal for use in care management because they missed more than 50% of the patients who will experience severe COPD exacerbations in the future, incorrectly projected many other patients to experience severe COPD exacerbations [20-22,53], used data unavailable in routine clinical practice [23-31,33,34,36,42-50,52], or were designed for patients who have different characteristics from typical patients with COPD [25-34]. In addition, most of these models predicted only inpatient stays for COPD. To better guide the use of care management, we need to predict both ED visits and inpatient stays for COPD, which only 2 of these models [34,36] do. In practice, once a model is deployed for care management, the prediction errors produced by the model would lead to degraded patient outcomes and unnecessary health care costs. Because of the large number of patients with COPD, even a small improvement in model accuracy coupled with appropriate preventive interventions could help improve outcomes and avoid many ED visits and inpatient stays for COPD every year.

### Objective

This study aims to develop a more accurate model to predict severe COPD exacerbations in the next year in patients with COPD. To be suitable for use in care management, the model should use data available in routine clinical practice and target all patients with COPD.

## Methods

### Ethics Approval and Study Design

The institutional review board of the University of Washington Medicine (UWM) approved this secondary analysis study on administrative and clinical data.

### Patient Population

In Washington state, the UWM is the largest academic health care system. The UWM enterprise data warehouse includes administrative and clinical data from 3 hospitals and 12 clinics. The patient cohort consisted of the patients with COPD who visited any of these facilities between 2011 and 2019. Using our prior method for identifying patients with COPD [54] that was adapted from the literature [55-58], we regarded a patient to have COPD if the patient was aged ≥40 years and met ≥1 of the 4 criteria listed in Textbox 1. When computing the data instances in any year, we excluded the patients who had no encounter at the UWM or died during that year. No other exclusion criterion was used.

**The 4 criteria used for identifying patients with chronic obstructive pulmonary disease.**

**Description of each of the 4 criteria**

- An outpatient visit diagnosis code of chronic obstructive pulmonary disease (International Classification of Diseases, Ninth Revision: 491.22, 491.21, 491.9, 491.8, 493.2x, 492.8, 496; International Classification of Diseases, Tenth Revision: J42, J41.8, J44.*, J43.*) followed by ≥1 prescription of long-acting muscarinic antagonist (aclidinium, glycopyrrolate, tiotropium, and umeclidinium) within 6 months
- ≥1 emergency department or ≥2 outpatient visit diagnosis codes of chronic obstructive pulmonary disease (International Classification of Diseases, Ninth Revision: 491.22, 491.21, 491.9, 491.8, 493.2x, 492.8, 496; International Classification of Diseases, Tenth Revision: J42, J41.8, J44.*, J43.*)
- ≥1 inpatient stay discharge having a principal diagnosis code of chronic obstructive pulmonary disease (International Classification of Diseases, Ninth Revision: 491.22, 491.21, 491.9, 491.8, 493.2x, 492.8, 496; International Classification of Diseases, Tenth Revision: J42, J41.8, J44.*, J43.*)
- ≥1 inpatient stay discharge having a principal diagnosis code of respiratory failure (International Classification of Diseases, Ninth Revision: 518.82, 518.81, 799.1, 518.84; International Classification of Diseases, Tenth Revision: J96.0*, J80, J96.9*, J96.2*, R09.2) and a secondary diagnosis code of acute chronic obstructive pulmonary disease exacerbation (International Classification of Diseases, Ninth Revision: 491.22, 491.21, 493.22, 493.21; International Classification of Diseases, Tenth Revision: J44.1, J44.0)

### Prediction Target (Also Known as the Outcome or the Dependent Variable)

**Figure 1:**
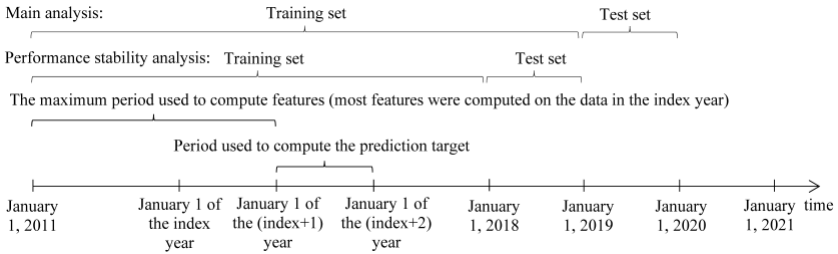
The periods used to partition the training and test sets and the periods used to compute the prediction target and the features for a patient and index year pair.

Given a patient with COPD who had ≥1 encounter at the UWM in a specific year (the index year), we used the patient’s data up to the last day of the year to predict the outcome of whether the patient would experience any severe COPD exacerbation, that is, any ED visit or inpatient stay with a principal diagnosis of COPD (International Classification of Diseases, Ninth Revision: 491.22, 491.21, 491.9, 491.8, 493.2x, 492.8, 496; International Classification of Diseases, Tenth Revision: J42, J41.8, J44.*, J43.*), in the next year (Figure 1).

### Data Set

We obtained a structured data set from the UWM enterprise data warehouse. This data set included administrative and clinical data relating to the patient cohort’s encounters at the 3 hospitals and 12 clinics of the UWM from 2011 to 2020.

### Features (Also Known as Independent Variables)

To improve model accuracy, we examined an extensive set of candidate features computed on the structured attributes in the data set. Table S1 of Multimedia Appendix 1 [3,18,28,30,50,59-83] shows these 278 candidate features coming from four sources: the known risk factors for COPD exacerbations [3,18,28,30,50,59-72], the features used in prior models to predict severe COPD exacerbations [20-53], the features that the clinician ZCL in our team suggested, and the features used in our prior models to predict asthma hospital encounters [84,85]. Asthma shares many similarities with COPD. Throughout this paper, whenever we mention the number of a given type of item (eg, medication) without using the word *distinct*, we count multiplicity.

Each input data instance to the predictive model contained 278 features, corresponded to a distinct patient and index year pair, and was used to predict the outcome of the patient in the next year. For this pair, the patient’s age was computed based on the age at the end of the index year. The patient’s primary care provider (PCP) was computed as the last recorded PCP of the patient by the end of the index year. The percentage of the PCP’s patients with COPD in the preindex year having severe COPD exacerbations in the index year was computed on the data in the preindex and index years. Using the data from 2011 to the index year, we computed 26 features: the number of years from the first encounter related to COPD in the data set, the type of the first encounter related to COPD in the data set, 7 allergy features, and 17 features related to the problem list. The other 251 features were computed on the data in the index year.

### Data Analysis

#### Data Preparation

Using the data preparation approach used in our papers [84,85], we identified the biologically implausible values, replaced them with null values, and normalized the data. As outcomes came from the next year, the data set had 9 years of effective data (2011-2019) over a time span of 10 years (2011-2020). To reflect future model use in clinical practice and to evaluate the impact of the COVID-19 pandemic on patient outcomes and model performance, we conducted two analyses:

1. Main analysis: we used the 2011-2018 data instances as the training set to train models and the 2019 data instances as the test set to assess model performance.
2. Performance stability analysis: we used the 2011-2017 data instances as the training set to train models and the 2018 data instances as the test set to assess model performance.

#### Classification Algorithms

We created machine learning classification models using Waikato Environment for Knowledge Analysis (WEKA; version 3.9) [86]. WEKA is a major open source software package for machine learning and data mining. It integrates many commonly used machine learning algorithms and feature selection techniques. We examined the 39 classification algorithms supported by WEKA and listed in the web-based multimedia appendix of our paper [84], as well as Extreme Gradient Boosting (XGBoost) [87] implemented in the XGBoost4J package [88]. XGBoost is a classification algorithm using an ensemble of decision trees. As XGBoost only takes numerical features, we converted categorical features to binary features through one-hot encoding. In the main analysis, we used the training set and our formerly published automatic machine learning model selection method [89] to automate the selection of the classification algorithm, feature selection technique, data balancing method to deal with imbalanced data, and hyperparameter values among all applicable ones. Compared with the Auto-WEKA automatic machine learning model selection method [90], our method achieved an average of 11% (SD 15%) reduction in model error rate and a 28-fold reduction in search time. In the performance stability analysis, we used the same classification algorithm, feature selection technique, and hyperparameter values as those used in the final model of the main analysis.

#### Performance Metrics

As shown in the formulas, the performance of the models was evaluated with respect to the following metrics: accuracy (Table 1); sensitivity, also known as recall; specificity; positive predictive value (PPV), also known as precision; negative predictive value (NPV); and area under the receiver operating characteristic curve (AUC):

Accuracy = (TP + TN) / (TP + TN + FP + FN) **(1)**

Sensitivity = TP / (TP + FN) **(2)**

Specificity = TN / (TN + FP) **(3)**

PPV = TP / (TP + FP) **(4)**

NPV = TN / (TN + FN) **(5)**

where TP stands for true positive, TN stands for true negative, FP stands for false positive, and FN stands for false negative.

**Table 1:** The confusion matrix.

Outcome class	Severe COPD^a^ exacerbations in the next year	No severe COPD exacerbation in the next year
Predicted severe COPD exacerbations in the next year	True positive	False positive
Predicted no severe COPD exacerbation in the next year	False negative	True negative

^a^COPD: chronic obstructive pulmonary disease.

We computed the 95% CIs of the performance measures using the bootstrapping method [91]. We obtained 1000 bootstrap samples from the test set and computed the model’s performance measures based on each bootstrap sample. This produced 1000 values for each performance metric. Their 2.5th and 97.5th percentiles provided the 95% CI of the corresponding performance measures. To depict the trade-off between sensitivity and specificity, we drew the receiver operating characteristic curve.

## Results

### Distributions of Data Instances and Bad Outcomes

The number of data instances increased over time. The proportion of data instances linked to bad outcomes remained relatively stable over time. The only exception was the sudden drop from 5.21% (369/7089) in 2018 to 2.42% (182/7529) in 2019 (Table 2), which resulted from the large drop in ED visits and inpatient stays for COPD in 2020 caused by the COVID-19 pandemic [92]. In the main analysis, 5.66% (2040/36,047) of the data instances in the training set and 2.42% (182/7529) of the data instances in the test set were linked to severe COPD exacerbations in the next year. In the performance stability analysis, 5.77% (1671/28,958) of the data instances in the training set and 5.21% (369/7089) of the data instances in the test set were linked to severe COPD exacerbations in the next year.

**Table 2:** The distributions of data instances and bad outcomes over time.

	Year								
	2011	2012	2013	2014	2015	2016	2017	2018	2019
Data instances, n	1848	2725	3204	4009	4875	5793	6504	7089	7529
Data instances linked to severe COPD^a^ exacerbations in the next year, n (%)	128 (6.93)	176 (6.46)	183 (5.71)	223 (5.56)	272 (5.58)	351 (6.06)	338 (5.2)	369 (5.21)	182 (2.42)

^a^COPD: chronic obstructive pulmonary disease.

### Patient Characteristics

Each patient and index year pair matched a data instance. For both the training set and the test set of the main analysis, when comparing the patient characteristic distributions between the data instances linked to severe COPD exacerbations in the next year and those linked to no severe COPD exacerbation in the next year, *P* values were computed using the chi-square 2-sample test and the Cochran–Armitage trend test [93] for categorical and numerical characteristics, respectively (Tables 3 and 4).

In the training set of the main analysis, most patient characteristics exhibited statistically significantly different distributions between the data instances linked to severe COPD exacerbations in the next year and those linked to no severe COPD exacerbation in the next year. Exceptions occurred on the patient characteristics of having prescriptions of inhaled corticosteroid, long-acting beta-2 agonist (LABA), and long-acting muscarinic antagonist (LAMA) combinations (*P*=.66); having prescriptions of phosphodiesterase-4 inhibitor (*P*=.06); presence of diabetes (*P*=.43); presence of eczema (*P*=.30); presence of lung cancer (*P*=.31); and presence of sinusitis (*P*=.61). In the test set of the main analysis, most patient characteristics exhibited statistically significantly different distributions between the data instances linked to severe COPD exacerbations in the next year and those linked to no severe COPD exacerbation in the next year. Exceptions occurred on the patient characteristics of having private insurance (*P*=.79); having prescriptions of LABA and LAMA combinations (*P*=.54); having prescriptions of inhaled corticosteroid, LABA, and LAMA combinations (*P*=.90); having prescriptions of phosphodiesterase-4 inhibitor (*P*=.27); presence of allergic rhinitis (*P*=.24); presence of anxiety or depression (*P*=.08); presence of congestive heart failure (*P*=.11); presence of diabetes (*P*=.95); presence of eczema (*P*=.08); presence of hypertension (*P*=.05); presence of lung cancer (*P*=.51); presence of obesity (*P*=.25); presence of sinusitis (*P*=.99); and presence of sleep apnea (*P*=.22).

**Table 3:** The patient characteristics of the data instances in the training set of the main analysis.

Patient characteristic			Data instances (N=36,047), n (%)		Data instances linked to severe COPD^a^ exacerbations in the next year (N=2040), n (%)		Data instances linked to no severe COPD exacerbation in the next year (N=34,007), n (%)		*P* value	
**Age (years)**										*<.001* ^b^
	40-65	18,793 (52.13)		1219 (59.75)		17,574 (51.68)		*<.001*		
	>65	17,254 (47.87)		821 (40.25)		16,433 (48.32)		*<.001*		
**Sex**										*<.001*
	Female	15,414 (42.76)		749 (36.72)		14,665 (43.12)		*<.001*		
	Male	20,633 (57.24)		1291 (63.28)		19,342 (56.88)		*<.001*		
**Race**										*<.001*
	American Indian or Alaska Native	713 (1.98)		26 (1.27)		687 (2.02)		*<.001*		
	Asian	2092 (5.8)		144 (7.06)		1948 (5.73)		*<.001*		
	Black or African American	4795 (13.3)		524 (25.69)		4271 (12.56)		*<.001*		
	Native Hawaiian or other Pacific Islander	184 (0.51)		8 (0.39)		176 (0.52)		*<.001*		
	White	27,447 (76.14)		1330 (65.2)		26,117 (76.8)		*<.001*		
	Other, unknown, or not reported	816 (2.27)		8 (0.39)		808 (2.37)		*<.001*		
**Ethnicity**										*<.001*
	Hispanic	857 (2.38)		53 (2.6)		804 (2.36)		*<.001*		
	Non-Hispanic	32,585 (90.39)		1941 (95.15)		30,644 (90.11)		*<.001*		
	Unknown or not reported	2605 (7.23)		46 (2.25)		2559 (7.53)		*<.001*		
**Smoking status**										*<.001*
	Current smoker	16,952 (47.03)		1089 (53.38)		15,863 (46.65)		*<.001*		
	Former smoker	7367 (20.44)		345 (16.91)		7022 (20.65)		*<.001*		
	Never smoker or unknown	11,728 (32.53)		606 (29.71)		11,122 (32.7)		*<.001*		
**Insurance**										
	Private	17,513 (48.58)		834 (40.88)		16,679 (49.05)		*<.001*		
	Public	29,598 (82.11)		1767 (86.62)		27,831 (81.84)		*<.001*		
	Self-paid or charity	1994 (5.53)		229 (11.23)		1765 (5.19)		*<.001*		
**Number of years from the first encounter related to** **COPD** **in the data set**										*<.001*
	≤3	30,315 (84.1)		1566 (76.76)		28,749 (84.54)		*<.001*		
	>3	5732 (15.9)		474 (23.24)		5258 (15.46)		*<.001*		
**COPD** **medication prescription**										
	ICS^c^	13,327 (36.97)		1119 (54.85)		12,208 (35.9)		*<.001*		
	SAMA^d^	9608 (26.65)		1042 (51.08)		8566 (25.19)		*<.001*		
	SABA^e^	22,549 (62.55)		1684 (82.55)		20,865 (61.36)		*<.001*		
	SABA and SAMA combination	7174 (19.9)		810 (39.71)		6364 (18.71)		*<.001*		
	LAMA^f^	10,243 (28.42)		1001 (49.07)		9242 (27.18)		*<.001*		
	LABA^g^	8904 (24.7)		842 (41.27)		8062 (23.71)		*<.001*		
	LABA and LAMA combination	426 (1.18)		40 (1.96)		386 (1.14)		*.001*		
	ICS and LABA combination	8326 (23.1)		782 (38.33)		7544 (22.18)		*<.001*		
	ICS, LABA, and LAMA combination	16 (0.04)		0 (0)		16 (0.05)		.66		
	Phosphodiesterase-4 inhibitor	94 (0.26)		10 (0.49)		84 (0.25)		.06		
	Systemic corticosteroid	11,293 (31.33)		1144 (56.08)		10,149 (29.84)		*<.001*		
**Comorbidity**										
	Allergic rhinitis	2445 (6.78)		174 (8.53)		2271 (6.68)		*.001*		
	Anxiety or depression	10,786 (29.92)		725 (35.54)		10,061 (29.59)		*<.001*		
	Asthma	4794 (13.3)		417 (20.44)		4377 (12.87)		*<.001*		
	Congestive heart failure	6063 (16.82)		495 (24.26)		5568 (16.37)		*<.001*		
	Diabetes	7623 (21.15)		446 (21.86)		7177 (21.1)		.43		
	Eczema	1558 (4.32)		98 (4.8)		1460 (4.29)		.30		
	Gastroesophageal reflux	7162 (19.87)		507 (24.85)		6655 (19.57)		*<.001*		
	Hypertension	18,361 (50.94)		1150 (56.37)		17,211 (50.61)		*<.001*		
	Ischemic heart disease	7420 (20.58)		486 (23.82)		6934 (20.39)		*<.001*		
	Lung cancer	794 (2.2)		52 (2.55)		742 (2.18)		.31		
	Obesity	3487 (9.67)		255 (12.5)		3232 (9.5)		*<.001*		
	Sinusitis	1382 (3.83)		83 (4.07)		1299 (3.82)		.61		
	Sleep apnea	3179 (8.82)		253 (12.4)		2926 (8.6)		*<.001*		

^a^COPD: chronic obstructive pulmonary disease.

^b^*P* value <.05 is italicized and signifies a statistically significant difference in the patient characteristic distributions.

^c^ICS: inhaled corticosteroid.

^d^SAMA: short-acting muscarinic antagonist.

^e^SABA: short-acting beta-2 agonist.

^f^LAMA: long-acting muscarinic antagonist.

^g^LABA: long-acting beta-2 agonist.

**Table 4:** The patient characteristics of the data instances in the test set of the main analysis.

Patient characteristic		Data instances (N=7529), n (%)	Data instances linked to severe COPD^a^ exacerbations in the next year (N=182), n (%)	Data instances linked to no severe COPD exacerbation in the next year (N=7347), n (%)	*P* value	
**Age (years)**						*<.001* ^b^
	40-65	3442 (45.72)	118 (64.8)	3324 (45.24)	*<.001*	
	>65	4087 (54.28)	64 (35.2)	4023 (54.76)	*<.001*	
**Sex**						*<.001*
	Female	3289 (43.68)	47 (25.8)	3242 (44.13)	*<.001*	
	Male	4240 (56.32)	135 (74.2)	4105 (55.87)	*<.001*	
**Race**						*<.001*
	American Indian or Alaska Native	156 (2.07)	5 (2.7)	151 (2.06)	*<.001*	
	Asian	439 (5.83)	7 (3.9)	432 (5.88)	*<.001*	
	Black or African American	896 (11.9)	57 (31.3)	839 (11.42)	*<.001*	
	Native Hawaiian or other Pacific Islander	53 (0.71)	2 (1.1)	51 (0.69)	*<.001*	
	White	5793 (76.94)	111 (61)	5682 (77.34)	*<.001*	
	Other, unknown, or not reported	192 (2.55)	0 (0)	192 (2.61)	*<.001*	
**Ethnicity**						*.03*
	Hispanic	188 (2.5)	3 (1.6)	185 (2.52)	*.03*	
	Non-Hispanic	7088 (94.14)	179 (98.4)	6909 (94.04)	*.03*	
	Unknown or not reported	253 (3.36)	0 (0)	253 (3.44)	*.03*	
**Smoking status**						*.03*
	Current smoker	3893 (51.71)	112 (61.5)	3781 (51.46)	*.03*	
	Former smoker	1267 (16.83)	25 (13.7)	1242 (16.91)	*.03*	
	Never smoker or unknown	2369 (31.47)	45 (24.7)	2324 (31.63)	*.03*	
**Insurance**						
	Private	4642 (61.65)	110 (60.4)	4532 (61.69)	.79	
	Public	6901 (91.66)	179 (98.4)	6722 (91.49)	*.002*	
	Self-paid or charity	540 (7.17)	41 (22.5)	499 (6.79)	*<.001*	
**Number of years from the first encounter related to COPD in the data set**						*<.001*
	≤3	5154 (68.46)	81 (44.5)	5073 (69.05)	*<.001*	
	>3	2375 (31.54)	101 (55.5)	2274 (30.95)	*<.001*	
**COPD** **medication prescription**						
	ICS^c^	2635 (35)	98 (53.8)	2537 (34.53)	*<.001*	
	SAMA^d^	1202 (15.96)	68 (37.4)	1134 (15.43)	*<.001*	
	SABA^e^	4241 (56.33)	158 (86.8)	4083 (55.57)	*<.001*	
	SABA and SAMA combination	1809 (24.03)	115 (63.2)	1694 (23.06)	*<.001*	
	LAMA^f^	2061 (27.37)	110 (60.4)	1951 (26.56)	*<.001*	
	LABA^g^	1760 (23.38)	77 (42.3)	1683 (22.91)	*<.001*	
	LABA and LAMA combination	400 (5.31)	12 (6.6)	388 (5.28)	.54	
	ICS and LABA combination	1804 (23.96)	75 (41.2)	1729 (23.53)	*<.001*	
	ICS, LABA, and LAMA combination	69 (0.92)	1 (0.5)	68 (0.93)	.90	
	Phosphodiesterase-4 inhibitor	26 (0.35)	2 (1.1)	24 (0.33)	.27	
	Systemic corticosteroid	2385 (31.68)	103 (56.6)	2282 (31.06)	*<.001*	
**Comorbidity**						
	Allergic rhinitis	410 (5.45)	14 (7.7)	396 (5.39)	.24	
	Anxiety or depression	2153 (28.6)	63 (34.6)	2090 (28.45)	.08	
	Asthma	1096 (14.56)	43 (23.6)	1053 (14.33)	*<.001*	
	Congestive heart failure	1412 (18.75)	43 (23.6)	1369 (18.63)	.11	
	Diabetes	1689 (22.43)	40 (22)	1649 (22.44)	.95	
	Eczema	258 (3.43)	11 (6)	247 (3.36)	.08	
	Gastroesophageal reflux	1443 (19.17)	47 (25.8)	1396 (19)	*.03*	
	Hypertension	3791 (50.35)	105 (57.7)	3686 (50.17)	.05	
	Ischemic heart disease	1658 (22.02)	54 (29.7)	1604 (21.83)	*.02*	
	Lung cancer	203 (2.7)	3 (1.6)	200 (2.72)	.51	
	Obesity	669 (8.89)	21 (11.5)	648 (8.82)	.25	
	Sinusitis	279 (3.71)	7 (3.8)	272 (3.7)	.99	
	Sleep apnea	915 (12.15)	28 (15.4)	887 (12.07)	.22	

^a^COPD: chronic obstructive pulmonary disease.

^b^*P* value <.05 is italicized and signifies a statistically significant difference in the patient characteristic distributions.

^c^ICS: inhaled corticosteroid.

^d^SAMA: short-acting muscarinic antagonist.

^e^SABA: short-acting beta-2 agonist.

^f^LAMA: long-acting muscarinic antagonist.

^g^LABA: long-acting beta-2 agonist.

### Classification Algorithm and Features Used in the Final Model

The XGBoost algorithm was chosen by our automatic machine learning model selection method [89]. As a tree-based algorithm, XGBoost handles missing values in the features naturally. As detailed in Hastie et al [94], XGBoost automatically calculates an importance value for each feature based on the feature’s apportioned contribution to the model. In the main analysis, the final model was created using XGBoost and the 229 features shown in descending order of their importance values in Table S2 of Multimedia Appendix 1. The other features contributed no extra predictive power and were automatically dropped by XGBoost.

### Model Performance in the Main Analysis

**Figure 2:**
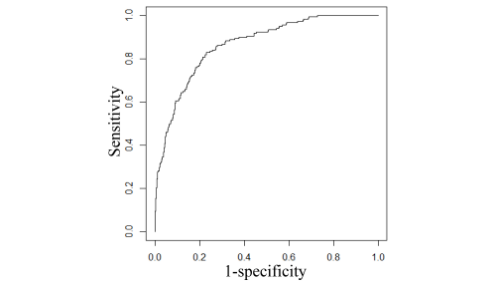
The receiver operating characteristic curve of the final model in the main analysis.

In the main analysis with the test set, the final model had an AUC of 0.866 (95% CI 0.838-0.892), as computed from the model’s receiver operating characteristic curve (Figure 2). The model’s performance measures varied with the cutoff threshold for binary classification (Table 5). When using the top 9.99% (752/7529) of the patients with the largest predicted risk to set the cutoff threshold for binary classification, the model had an accuracy of 90.33% (6801/7529; 95% CI 89.61%-91.01%), a sensitivity of 56.6% (103/182; 95% CI 49.2%-64.2%), a specificity of 91.17% (6698/7347; 95% CI 90.51%-91.83%), a PPV of 13.7% (103/752; 95% CI 11.2%-16.2%), and an NPV of 98.83% (6698/6777; 95% CI 98.55%-99.08%), as computed from the corresponding confusion matrix of the model (Table 6).

Recall that 27 candidate features were computed on ≥2 years of data. When we ignored these features and considered only those computed with the data in the index year, the model’s AUC dropped from 0.866 to 0.859 (95% CI 0.834-0.884). The top 19 features shown in Table S2 of Multimedia Appendix 1 have importance values ≥1%. When using only these features, the model’s AUC dropped from 0.866 to 0.862 (95% CI 0.837-0.887). In this case, when using the top 9.99% (752/7529) of the patients with the largest predicted risk to set the cutoff threshold for binary classification, the model had an accuracy of 90.25% (6795/7529; 95% CI 89.56%-90.9%), a sensitivity of 54.9% (100/182; 95% CI 47.8%-61.9%), a specificity of 91.13% (6695/7347; 95% CI 90.43%-91.78%), a PPV of 13.3% (100/752; 95% CI 10.9%-15.7%), and an NPV of 98.79 (6695/6777; 95% CI 98.52%-99.06%).

**Table 5:** In the main analysis, the performance measures of the final model with respect to using varying cutoff thresholds for binary classification.

Top percentage of patients with the largest predicted risk (%)	Accuracy (N=7529), n (%)	Sensitivity (N=182), n (%)	Specificity (N=7347), n (%)	Positive predictive value		Negative predictive value	
				n (%)	N	n (%)	N
1	7336 (97.4)	32 (17.6)	7304 (99.4)	32 (42.7)	75	7304 (98)	7454
2	7299 (96.9)	51 (28)	7248 (98.7)	51 (34)	150	7248 (98.2)	7379
3	7236 (96.1)	57 (31.3)	7179 (97.7)	57 (25.3)	225	7179 (98.3)	7304
4	7170 (95.2)	62 (34.1)	7108 (96.7)	62 (20.6)	301	7108 (98.3)	7228
5	7111 (94.4)	70 (38.5)	7041 (95.8)	70 (18.6)	376	7041 (98.4)	7153
6	7062 (93.8)	83 (45.6)	6979 (95)	83 (18.4)	451	6979 (98.6)	7078
7	6994 (92.9)	87 (47.8)	6907 (94)	87 (16.5)	527	6907 (98.6)	7002
8	6927 (92)	91 (50)	6836 (93)	91 (15.1)	602	6836 (98.7)	6927
9	6860 (91.1)	95 (52.2)	6765 (92.1)	95 (14)	677	6765 (98.7)	6852
10	6801 (90.3)	103 (56.6)	6698 (91.2)	103 (13.7)	752	6698 (98.8)	6777
15	6458 (85.8)	120 (65.9)	6338 (86.3)	120 (10.6)	1129	6338 (99)	6400
20	6118 (81.3)	138 (75.8)	5980 (81.4)	138 (9.2)	1505	5980 (99.3)	6024
25	5767 (76.6)	151 (83)	5616 (76.4)	151 (8)	1882	5616 (99.5)	5647

**Table 6:** The confusion matrix of the final model in the main analysis when using the top 9.99% (794/7944) of the patients with the largest predicted risk to set the cutoff threshold for binary classification.

Outcome class	Severe COPD^a^ exacerbations in the next year	No severe COPD exacerbation in the next year
Predicted severe COPD exacerbations in the next year	103	649
Predicted no severe COPD exacerbation in the next year	79	6698

^a^COPD: chronic obstructive pulmonary disease.

### Performance Stability Analysis

The final model in the main analysis and the model in the performance stability analysis had relatively similar performance (Table 7).

**Table 7:** The performance of the final model in the main analysis and the model in the performance stability analysis.

Performance measure	Final model in the main analysis^a^			Model in the performance stability analysis^b^	
	n (%; 95% CI)	N	n (%; 95% CI)		N
Accuracy	6801 (90.3; 89.6-91.0)	7529	6354 (89.6; 88.9-90.3)		7089
Sensitivity	103 (56.6; 49.2-64.2)	182	171 (46.3; 40.9-51.5)		369
Specificity	6698 (91.2; 90.5-91.8)	7347	6183 (92; 91.4-92.7)		6720
Positive predictive value	103 (13.7; 11.2-16.2)	752	171 (24.2; 20.8-27.2)		708
Negative predictive value	6698 (98.8; 98.6-99.1)	6777	6183 (96.9; 96.4-97.3)		6381

^a^Area under the receiver operating characteristic curve of 0.866 (95% CI 0.838-0.892).

^b^Area under the receiver operating characteristic curve of 0.847 (95% CI 0.828-0.864).

## Discussion

### Principal Findings

We created a machine learning model to predict severe COPD exacerbations in the next year in patients with COPD. The model had a higher AUC than the formerly published AUC of every prior model for predicting severe COPD exacerbations in the next year [20,25,27,28,30,33,35-43,46-49,51] (Tables 8 and 9). After improving our model’s performance measures further (eg, by adding features extracted from clinical notes) and using our recently published automatic explanation method [95] to automatically explain the model’s predictions, our model could be used as a decision support tool to advise the use of care management for patients with COPD and at high risk to improve outcomes.

In Table S2 of Multimedia Appendix 1, many of the top 19 features match the published (risk) factors that were highly correlated with COPD exacerbations, such as prior COPD exacerbations [18,60], prior health care encounters related to COPD [28,50], COPD medication use [50], BMI [70], peripheral capillary oxygen saturation [28], and heart rate [71].

We examined 278 candidate features, 82.4% (229/278) of which were used in the final model. Many omitted features are correlated with the outcome, but they provided no extra predictive power on the UWM data set beyond the 229 features used in the final model.

The prevalence rate of severe COPD exacerbations had a sudden drop in 2019. Despite this drop, our model still showed reasonably robust performance over time. This is desired for clinical decision support.

**Table 8:** A comparison of our final model and several prior models to predict severe chronic obstructive pulmonary disease (COPD) exacerbations in patients with COPD (Part 1).

Model	Data	Number of data instances	Prediction target (outcome)	Length of the period used to compute the outcome	Prevalence rate of the poor outcome (%)	Number of features checked	Classification algorithm	Sensitivity (%)	Specificity (%)	PPV^a^ (%)	NPV^b^ (%)	AUC^c^
Our final model	Administrative and clinical	43,576	ED^d^ visit or inpatient stay for COPD	1 year	5.1	278	XGBoost^e^	56.6	91.17	13.7	98.83	0.866
Annavarapu et al [20]	Administrative	45,722	Inpatient stay for COPD	1 year	11.63	103	Logistic regression	17.3	97.5	48.1	90	0.77
Tavakoli et al [21]	Administrative	222,219	Inpatient stay for COPD	2 months	1.02	83	Gradient boosting	23	98	—^f^	—	0.820
Samp et al [22]	Administrative	478,772	Inpatient stay for COPD	6 months	2.2	101	Logistic regression	17.6	96.6	—	—	—
Thomsen et al [23]	Research	6574	Two or more exacerbations (medication change or inpatient stay for COPD)	1-7 years	6.4	11	Logistic regression	—	—	18	96	0.73
Orchard et al [24]	Research	57,150	Inpatient stay for COPD	1 day	0.1	153	Neural network	80	60	—	—	0.740
Suetomo et al [25]	Research	123	Inpatient stay for COPD	1 year	12.2	18	Logistic regression	53	49	—	—	0.79
Lee et al [26]	Research and clinical	545	Medication change, ED visit, or inpatient stay for COPD	6 months	46	10	Logistic regression	52	69	—	—	0.63
Faganello et al [27]	Research	120	Outpatient, inpatient, or ED encounter for COPD	1 year	50	16	Logistic regression	58.3	73.3	—	—	0.686
Alcázar et al [28]	Research	127	Inpatient stay for COPD	1 year	39.4	9	Logistic regression	76.2	77.3	61.5	87.2	0.809
Bertens et al [29]	Research and clinical	1033	Medication change or inpatient stay for COPD	2 years	28.3	7	Logistic regression	—	—	—	—	0.66
Miravitlles et al [30]	Research and clinical	713	Inpatient stay for COPD	1 year	22.2	7	Logistic regression	—	—	—	—	0.582
Make et al [31]	Research	3141	Medication change, ED visit, or inpatient stay for COPD	6 months	—	38	Logistic regression	—	—	—	—	0.67
Montserrat-Capdevila et al [32]	Administrative and clinical	2501	Inpatient stay for COPD	3 years	32.5	17	Logistic regression	—	—	—	—	0.72
Kerkhof et al [33]	Research and clinical	16,565	Two or more exacerbations (medication change, ED visit, or inpatient stay for COPD)	1 year	19.6	22	Logistic regression	—	—	—	—	0.735
Chen et al [34]	Research	1711	ED visit or inpatient stay for COPD	5 years	30.6	14	Cox proportional hazard regression	—	—	—	—	0.74
Yii et al [35]	Administrative and clinical	237	Inpatient stay for COPD	1 year	1.41 per patient year	31	Negative binomial regression	—	—	—	—	0.789

^a^PPV: positive predictive value.

^b^NPV: negative predictive value.

^c^AUC: area under the receiver operating characteristic curve.

^d^ED: emergency department.

^e^XGBoost: Extreme Gradient Boosting.

^f^The performance measure is unreported in the initial paper describing the model.

**Table 9:** A comparison of our final model and several prior models to predict severe chronic obstructive pulmonary disease (COPD) exacerbations in patients with COPD (Part 2).

Model	Data	Number of data instances	Prediction target (outcome)	Length of the period used to compute the outcome	Prevalence rate of the poor outcome (%)	Number of features checked	Classification algorithm	Sensitivity (%)	Specificity (%)	PPV^a^ (%)	NPV^b^ (%)	AUC^c^
Our final model	Administrative and clinical	43,576	ED^d^ visit or inpatient stay for COPD	1 year	5.1	278	XGBoost^e^	56.6	91.17	13.7	98.83	0.866
Adibi et al [36]	Research	2380	ED visit or inpatient stay for COPD	1 year	0.29 per year	13	Mixed effect logistic	—^f^	—	—	—	0.77
Stanford et al [37]	Administrative	258,668	Inpatient stay for COPD	1 year	8.5	30	Logistic regression	—	—	—	—	0.749
Stanford et al [38]	Administrative	223,824	Inpatient stay for COPD	1 year	6.63	30	Logistic regression	—	—	—	—	0.711
Stanford et al [39]	Administrative	92,496	Inpatient stay for COPD	1 year	—	30	Logistic regression	—	—	—	—	0.801
Stanford et al [40]	Administrative	60,776	Inpatient stay for COPD	1 year	19.16	8	Logistic regression	—	—	—	—	0.742
Jones et al [41]	Clinical	375	Inpatient stay for COPD	1 year	—	4	Index	—	—	—	—	0.755
Jones et al [42]	Research and clinical	7105	Inpatient stay for COPD	1 year	—	8	Negative binomial regression	—	—	—	—	0.64
Fan et al [43]	Research	3282	Inpatient stay for COPD	1 year	4.3	23	Logistic regression	—	—	—	—	0.706
Moy et al [44]	Research and clinical	167	Inpatient stay for COPD	4-21 months	32.9	6	Negative binomial regression	—	—	—	—	0.69
Briggs et al [45]	Research	8802	Inpatient stay for COPD	6 months to 3 years	9	13	Cox proportional hazard regression	—	—	—	—	0.71
Lange et al [46]	Administrative and research	6628	Medication change or inpatient stay for COPD	1 year	4.8	3	GOLD^g^ stratification	—	—	—	—	0.7
Abascal-Bolado et al [47]	Research and clinical	493	Inpatient stay for COPD	1 year	—	8	Classification and regression tree	—	—	—	—	0.70
Blanco-Aparicio et al [48]	Research	100	ED visit for COPD	1 year	21	12	Logistic regression	—	—	—	—	0.651
Yoo et al [49]	Research and clinical	260	Medication change, ED visit, or inpatient stay for COPD	1 year	40.8	17	Logistic regression	—	—	—	—	0.69
Niewoehner et al [50]	Research and clinical	1829	Inpatient stay for COPD	6 months	8.3	27	Cox proportional hazard regression	—	—	—	—	0.73
Austin et al [51]	Administrative	638,926	COPD-related inpatient stay	1 year	—	34	Logistic regression	—	—	—	—	0.778
Marin et al [52]	Research	275	Inpatient stay for COPD	6 months to 8 years	—	4	Logistic regression	86	73	—	—	0.88
Marin et al [52]	Research	275	ED visit for COPD	6 months to 8 years	—	4	Logistic regression	58	87	—	—	0.78
Ställberg et al [53]	Administrative and clinical	7823	COPD-related inpatient stay	10 days	—	>4000	XGBoost	16	—	11	—	0.86

^a^PPV: positive predictive value.

^b^NPV: negative predictive value.

^c^AUC: area under the receiver operating characteristic curve.

^d^ED: emergency department.

^e^XGBoost: Extreme Gradient Boosting.

^f^The performance measure is unreported in the initial paper describing the model.

^g^GOLD: Global Initiative for Chronic Obstructive Lung Disease.

### Comparison With Prior Work

Researchers formerly created several models to predict severe COPD exacerbations in patients with COPD [20-53]. Tables 8 and 9 present comparisons between our final model and these models, which include all related models listed in the systematic reviews by Guerra et al [96] and Bellou et al [97] as well as several recent models that were published after the reviews. Our final model predicted severe COPD exacerbations in the next year. Every prior model for predicting severe COPD exacerbations in the next year had an AUC ≤0.809, that is, at least 0.057 lower than that of our final model. Compared with the prior models for predicting severe COPD exacerbations other than the model developed by Ställberg et al [53], our final model used more extensive features with predictive power, which helped improve model performance.

Our final model’s prediction target covered both future ED visits and future inpatient stays for COPD, which we want to use care management to prevent. Among all prior models, only 2 [34,36] had prediction targets covering both future ED visits and future inpatient stays for COPD. Most of the prior models predicted either only future ED visits [48,52] or only future inpatient stays for COPD [20-22,24,25,28,30,32,35,37-45, 47,50-52]. This would be insufficient for preventing both future ED visits and future inpatient stays for COPD. The other prior models [23,26,27,29,31,33,46,49] had prediction targets covering both moderate and severe COPD exacerbations, with moderate COPD exacerbations typically referring to COPD medication change such as the use of systemic corticosteroids. These prediction targets were not specific enough for identifying patients at the highest risk for care management because a care management program can host only a small portion of patients [17].

To make it suitable for use in daily clinical practice, our final model was built on routinely available administrative and clinical data. In comparison, the models developed by several other research groups [23-31,33,34,36,42-50,52] used research data, some of which are unavailable in usual clinical practice. Thus, these models would be unsuitable for daily clinical use.

Our predictive model was developed to guide COPD care management’s enrollment decisions and to prevent severe COPD exacerbations. To give enough lead time for preventive interventions to be effective and to use precious care management resources well, we chose severe COPD exacerbation in the next year as the prediction target. In comparison, the model developed by Orchard et al [24] predicted inpatient stays for COPD on the next day. If a patient will incur an inpatient stay for COPD tomorrow, intervening starting from today could be too late to avoid the inpatient stay. At present, we are aware of no published conclusion on how long it will take for any intervention to be effective at preventing severe COPD exacerbations. In the studies by Longman et al [98] and Johnston et al [99], several clinicians had expressed the opinion that it could take as long as 3 months for any intervention to be effective at preventing inpatient stays for a chronic, ambulatory care–sensitive condition. Our final model will have a different clinical use from the models that make short-term predictions. Foreseeing a severe COPD exacerbation in the next 12 months would be useful for identifying and personalizing medium-term interventions and maintenance therapies to change the course of the disease. In comparison, foreseeing a severe COPD exacerbation in the next 1 or few days can be useful for deciding acute management approaches to improve outcomes, such as preemptive hospitalization of the patient to avoid more severe adverse outcomes, but would be inadequate for trying to improve the course of the disease in a short amount of time. In fact, treatment approaches proven to be effective at reducing severe COPD exacerbations are usually not indicated for acute management.

Marin et al [52] built a model to predict inpatient stays for COPD in up to the next 8 years with an AUC of 0.88 and a separate model to predict ED visits for COPD in up to the next 8 years with an AUC of 0.78. An inpatient stay or an ED visit that will happen several years later is too remote to be worth using precious care management resources now to prevent.

For the patients with COPD who will have severe COPD exacerbations in the future, sensitivity is the proportion of patients whom the model identifies. The difference in sensitivity could greatly affect hospital use. Our final model’s sensitivity is higher than the sensitivities achieved by the models developed by several other research groups [20-22,25,26,53]. Compared with our final model, the models developed by Orchard et al [24], Faganello et al [27], and Alcázar et al [28] each reached a higher sensitivity at the price of a much lower specificity. For each of these 3 models, if we adjust the cutoff threshold for binary classification and make our final model have the same specificity as that model, our final model would achieve a higher sensitivity than that model. More specifically, at a specificity of 60.02% (4410/7347), our final model achieved a sensitivity of 90.1% (164/182), whereas the model developed by Orchard et al [24] achieved a sensitivity of 80%. At a specificity of 73.3% (5385/7347), our final model achieved a sensitivity of 84.1% (153/182), whereas the model developed by Faganello et al [27] achieved a sensitivity of 58.3%. At a specificity of 77.34% (5682/7347), our final model achieved a sensitivity of 81.9% (149/182), whereas the model developed by Alcázar et al [28] achieved a sensitivity of 76.2%.

The prevalence rate of poor outcomes has a large impact on any model’s PPV [100]. On our data set, where this prevalence rate is approximately 5%, our final model reached a PPV of <14%. In comparison, on a data set where this prevalence rate is 11.63%, the model developed by Annavarapu et al [20] reached a PPV of 48.1%. On a data set where this prevalence rate is 6.4%, the model developed by Thomsen et al [23] reached a PPV of 18%. On a data set where this prevalence rate is 39.4%, the model developed by Alcázar et al [28] reached a PPV of 61.5%. In all 3 cases, the higher prevalence rates of poor outcomes permitted the PPV to be larger.

Our data set is imbalanced, with only a small portion of patients to have severe COPD exacerbations in the next year. For imbalanced data sets, the area under the precision–recall curve (AUPRC) is a better measure of overall model performance than the AUC [101]. The AUPRC was reported for only the model developed by Ställberg et al [53] among all the prior models. Although the model developed by Ställberg et al [53] had an AUC of 0.86, which is only slightly lower than that of our final model, our final model had an AUPRC of 0.24 (95% CI 0.18-0.31) that is 3 times as large as the 0.08 AUPRC of that model. In addition, that model predicted COPD-related inpatient stays, for which COPD can be any of the diagnoses, in the next 10 days. If a patient will incur an inpatient stay in the next 10 days, intervening starting from today could be too late to avoid the inpatient stay. In comparison, our final model predicted ED visits or inpatient stays with a principal diagnosis of COPD in the next year, allowing more lead time for preventive interventions to be effective.

### Considerations for Future Clinical Use

Our final model reached an AUC that is larger than every AUC formerly reported in the literature for predicting severe COPD exacerbations in the next year. Despite having a relatively low PPV, our final model could still benefit health care for 3 reasons.

First, health care systems such as the UWM and Intermountain Healthcare use proprietary models, which have similar performance to the formerly published models, to allocate COPD care management resources. Our final model had a higher AUC than all formerly reported AUCs for predicting severe COPD exacerbations in the next year. Hence, although we plan to investigate using various techniques to further improve model performance in the future, we think it is already worth considering using our final model to replace the proprietary models currently being used at health care systems such as the UWM for COPD care management.

Second, we set the cutoff threshold for binary classification at the top 9.99% (752/7529) of the patients with the largest predicted risk. In this case, a perfect model would achieve the theoretically maximum possible PPV of 24.2% (182/752). Our final model’s PPV is 56.6% (103/182) of the theoretically maximum possible PPV. In other words, our final model captured 56.6% (103/182) of the patients with COPD who would have severe COPD exacerbations in the next year. If we change the cutoff threshold to the top 25% of the patients with the largest predicted risk, the final model would capture 83% (151/182) of the patients with COPD who would have severe COPD exacerbations in the next year.

Third, a PPV at the level of our final model’s PPV is suitable for identifying patients with COPD and at high risk for low-cost preventive interventions such as arranging a nurse to further follow up with the patient through phone calls, teaching the patient to correctly use a COPD inhaler, teaching the patient the correct use of a peak flow meter to self-monitor symptoms at home, and enrolling the patient in a home-based pulmonary rehabilitation program [102].

Our final model used 229 features. To ease clinical deployment, we could reduce features, for example, to the top 19 with importance values ≥1%. A feature’s importance value differs across health care systems. If conditions permit, we should use a data set from the target health care system to compute the features’ importance values and decide which features to retain.

Our final model was based on XGBoost [87], which leverages the hyperparameter scale_pos_weight to balance the weights of the 2 outcome classes in our data set [103]. The scale_pos_weight hyperparameter was set by our automatic model selection method [89] to a nondefault value to maximize our final model’s AUC [104]. This caused the side effect of greatly increasing our model’s predicted probabilities of having future severe COPD exacerbations to values much larger than the true probabilities [103]. However, it does not affect our ability to identify the top portion of the patients with the largest predicted risk for preventive interventions. If preferred, we could forgo the balancing by keeping scale_pos_weight at its default value 1. In this case, our model’s AUC would drop by 0.003 to 0.863 (95% CI 0.835-0.888), which is still larger than every formerly published AUC for predicting severe COPD exacerbations in the next year.

### Limitations

This study includes several limitations that are worth future work.

First, this study used solely structured data. It is worth considering performing natural language processing to extract features from unstructured clinical notes to improve model performance. A model with higher performance can be used to better facilitate COPD care management.

Second, this study used age, diagnosis codes, and medication data to identify patients with COPD and used diagnosis codes and encounter information to define the prediction target. One can use age, diagnosis codes, and medication data to identify patients with COPD reasonably well [56]; yet, diagnosis codes were shown to have a low sensitivity in capturing inpatient stays for COPD [105]. Our predictive model is likely to perform poorly at finding those patients who would experience only future inpatient stays for COPD that are not captured by our current definition of the prediction target. We expect that this will not greatly affect our predictive model’s usefulness for facilitating COPD care management. On the basis of our current definition of the prediction target, >5% of the patients in our data set had severe COPD exacerbations in the following year. If fully captured by the predictive model, these patients would have already exceeded the service capacity of a typical care management program, which can take ≤3% of the patients [17]. In the future, one could consider adding both medication data and information extracted from clinical notes through natural language processing to better capture inpatient stays for COPD.

Third, this study used non–deep learning classification algorithms. Deep learning has improved model performance for many clinical predictive modeling tasks [106-111]. It is worth investigating whether using deep learning can improve model performance for predicting severe COPD exacerbations.

Fourth, this study used data from a single health care system: the UWM. It is worth evaluating our model’s generalizability to other health care systems. We are working on obtaining a data set of patients with COPD from Intermountain Healthcare for this purpose [112].

Fifth, our data set contained no information on UWM patients’ health care use at other health care systems. It is worth evaluating how our model’s performance would change if data on UWM patients’ health care use at other health care systems are available.

### Conclusions

This work improved the state of the art of predicting severe COPD exacerbations in patients with COPD. In particular, our final model had a higher AUC than every formerly published model AUC on predicting severe COPD exacerbations in the next year. After improving our model’s performance measures further and using our recently published automatic explanation method [95] to automatically explain the model’s predictions, our model could be used in a decision support tool to guide the use of care management for patients with COPD and at high risk to improve outcomes.

## Appendix

Multimedia Appendix 1 The candidate features and the features used in the final model in the main analysis and their importance values.

## Abbreviations

AUC: area under the receiver operating characteristic curve
AUPRC: area under the precision–recall curve
COPD: chronic obstructive pulmonary disease
ED: emergency department
LABA: long-acting beta-2 agonist
LAMA: long-acting muscarinic antagonist
NPV: negative predictive value
PCP: primary care provider
PPV: positive predictive value
UWM: University of Washington Medicine
WEKA: Waikato Environment for Knowledge Analysis
XGBoost: Extreme Gradient Boosting
