# Supplementary material for: Developing a Machine Learning Model to Predict Severe Chronic Obstructive Pulmonary Disease Exacerbations: Retrospective Cohort Study
Source: J Med Internet Res. 2022 Jan 6;24(1):e28953. doi: 10.2196/28953 (PMC8778560; doi:10.2196/28953)
Supplement: Multimedia Appendix 1 [file jmir_v24i1e28953_app1.pdf]

**Appendix**

**Table S1**. The candidate features.

| Feature category | Features | Notes |
|---|---|---|
| Patient demographics | Age [30,50,59]; gender; marital status (married, single, partnered, divorced, widowed, or separated); race; ethnicity (Hispanic or non-Hispanic); and language. | Acceptable range:<br>Age: 40-122 years [102].<br>Number of features: 6. |
| Laboratory test | Minimum Alpha-1 antitrypsin (A1AT) level; maximum A1AT level; whether the minimum A1AT level is abnormally low; minimum arterial oxygen saturation ($SaO_2$) level [69]; maximum arterial partial pressure of carbon dioxide ($PaCO_2$) level; minimum $PaCO_2$ level; maximum arterial partial pressure of oxygen ($PaO_2$) level; minimum $PaO_2$ level; maximum blood eosinophil count; maximum percentage of blood eosinophils; maximum blood neutrophil count [65,66]; maximum percentage of blood neutrophils; maximum C-reactive protein (CRP) level [64]; whether the maximum CRP level is abnormally high; maximum hematocrit (Hct) level; minimum Hct level [67]; whether the maximum Hct level is abnormally high; whether the minimum Hct level is abnormally low; maximum hemoglobin A1c (HbA1c) level; maximum hemoglobin (Hgb) level [68]; minimum Hgb level; whether the maximum Hgb level is abnormally high; whether the minimum Hgb level is abnormally low; whether an immunoglobulin E (IgE) test was performed; maximum total serum IgE level; whether the maximum total serum IgE level is abnormally high; maximum red blood cell count [63]; maximum white blood cell count [18,60]; no. of laboratory tests; no. of days from the last laboratory test; and no. of laboratory tests having abnormal results. | Number of features: 31. |
| Vital sign | Maximum body mass index (BMI) [70]; the relative change of BMI = (the last logged BMI / the first logged BMI - 1) × 100%; maximum diastolic blood pressure; average diastolic blood pressure; maximum heart rate [71]; average heart rate; maximum height; minimum peak expiratory flow [61]; average peak expiratory flow; minimum peripheral capillary oxygen saturation ($SpO_2$) [28]; average $SpO_2$; maximum respiratory rate; average respiratory rate; maximum systolic blood pressure; average systolic blood pressure; maximum temperature; average temperature; and the relative change of weight = (the last logged weight / the first logged weight - 1) × 100%. | Acceptable ranges:<br>Weight: 0.26-635 kilograms [103,104]; height: 0.24-2.72 meters [105,106]; BMI: 7.5-204.0 [107,108]; systolic and diastolic blood pressure: 0-300 mm Hg; heart rate: 30-300 beats/minute; respiratory rate: 0-120; temperature: 80-110 ˚F; $SpO_2$: 0-100; and peak expiratory flow: 0-700 liters/minute.<br>Number of features: 18. |
| Spirometry | Whether a spirometry was performed; average forced expiratory volume in 1 second ($FEV_1$) [61]; minimum $FEV_1$; average $FEV_1$ percent predicted; minimum $FEV_1$ percent predicted; average forced vital capacity (FVC); minimum FVC; average $FEV_1$/FVC ratio; minimum $FEV_1$/FVC ratio; whether any $FEV_1$ percent predicted is <80% with a normal $FEV_1$/FVC ratio (preserved ratio impaired spirometry, PRISm); and the highest GOLD stage of COPD [3,18,30,50,60]. | $FEV_1$ percent predicted was computed using the Hankinson's prediction equation [109-111].<br>Number of features: 11. |
| Diagnosis | Computed based on International Classification of Diseases, Ninth Revision (ICD-9) and International Classification of Diseases, Tenth Revision (ICD-10) diagnosis codes: No. of ICD-9 and ICD-10 diagnosis codes; no. of chronic obstructive pulmonary disease (COPD) diagnoses; no. of primary or principal COPD diagnoses; no. of diagnoses of acute COPD exacerbation; no. of years from the first encounter related to COPD [50]; whether the last COPD diagnosis is a primary or principal diagnosis; no. of days from the last COPD diagnosis; no. of days from the last diagnosis of acute COPD exacerbation; no. of diagnoses of | Number of features: 62. |

| | | |
|---|---|---|
| | noncompliance with medication regimen; acquired immunodeficiency syndrome; allergic rhinitis; Alzheimer's or Parkinson's disease; anaphylaxis; anxiety or depression [72]; asthma; breathing abnormality like dyspnea; bronchopulmonary dysplasia; cirrhosis; cerebrovascular disease; congestive heart failure [30]; cystic fibrosis; decreased tone; dementia; diabetes without chronic complication [30]; diabetes with chronic complication; eczema; esophagitis; folate deficiency; gastroesophageal reflux [18]; gastrointestinal bleeding; gastrointestinal obstruction; gastrostomy tube; hypertension; immunoglobulin A (IgA) deficiency; increased tone; inflammatory bowel disease; ischemic heart disease [30]; lung cancer; malignancy; mental disorder; metastatic solid tumor; mild liver disease; moderate or severe liver disease; myocardial infarction; obesity; paraplegia or hemiplegia; peptic ulcer disease; peripheral vascular disease [50]; pneumonia; pregnancy; psoriasis; renal disease [30]; rheumatic disease; sleep apnea; substance use; upper respiratory tract infection; vasculitis; vitamin D deficiency; and vocal cord dysfunction.<br>Computed based on ICD-9 and ICD-10 procedure and diagnosis codes: Tracheostomy.<br>Computed based on Current Procedural Terminology (CPT) and Healthcare Common Procedure Coding System (HCPCS) procedure codes: Cataract; and sinusitis. | |
| Problem list | No. of active problems; no. of active problems of COPD; no. of active problems of COPD exacerbation; no. of active problems of anxiety or depression; no. of active problems of asthma; no. of active problems of asthma with (acute) exacerbations; no. of active problems of gastroesophageal reflux disease; no. of active problems of congestive heart failure; no. of active problems of diabetes; no. of active problems of dyspnea; no. of active problems of hypertension; no. of active problems of obesity; no. of active problems of rhinitis; no. of active problems of sleep apnea; no. of active problems about smoking; no. of active problems of wheezing; and the priority of the last active problem of COPD. | Number of features: 17. |
| Medication | Total no. of COPD medications ordered; no. of COPD medication orders; total no. of distinct COPD medications ordered; total no. of COPD medication refills allowed; total no. of units of COPD medications ordered; no. of COPD reliever orders; total no. of medications in COPD reliever orders; total no. of distinct medications in COPD reliever orders; total no. of refills allowed for COPD relievers; total no. of units of COPD relievers ordered; no. of COPD controller orders; total no. of medications in COPD controller orders; total no. of distinct medications in COPD controller orders; total no. of refills allowed for COPD controllers; total no. of units of COPD controllers ordered; whether a nebulizer was used; no. of nebulizer medication orders; total no. of medications in nebulizer medication orders; total no. of distinct medications in nebulizer medication orders; total no. of refills allowed for nebulizer medications; total no. of units of nebulizer medications ordered; whether a spacer was used; no. of medication orders; total no. of medications ordered; total no. of distinct medications ordered; total no. of units of medications ordered; total no. of medication refills allowed; total no. of short-acting muscarinic antagonists (SAMA) ordered; total no. of refills allowed for SAMA; total no. of units of SAMA ordered; total no. of inhaled corticosteroids (ICS) ordered; total no. of refills allowed for ICS; total no. of units of ICS ordered; total no. of short-acting beta-2 agonists (SABA) ordered; total no. of refills allowed for SABA; total no. of units of SABA ordered; total no. of | COPD medication categories [3,112]:<br>• Short-term relievers: systemic corticosteroid [50]; short-acting muscarinic antagonist (SAMA) [50]; short-acting beta-2 agonist (SABA); SABA and SAMA combination; and mucolytic agent.<br>• Long-term controllers: inhaled corticosteroid (ICS); long-acting muscarinic antagonist (LAMA); long-acting beta-2 agonist (LABA); LABA and LAMA combination; ICS and LABA combination; ICS, LABA, and LAMA combination; methylxanthine; anti-interleukin-5; anti-interleukin-5 receptor; and phosphodiesterase-4 inhibitor (PDE-4).<br>Number of features: 52. |

| | | |
|---|---|---|
| | systemic corticosteroid ordered; total no. of refills allowed for systemic corticosteroids; total no. of units of systemic corticosteroids ordered; total no. of long-acting beta-2 agonists (LABA) ordered; total no. of refills allowed for LABA; total no. of units of LABA ordered; total no. of long-acting muscarinic antagonists (LAMA) ordered; total no. of refills allowed for LAMA; total no. of units of LAMA ordered; total no. of phosphodiesterase-4 inhibitors (PDE-4) ordered; total no. of refills allowed for PDE-4; total no. of units of PDE-4 ordered; total no. of ICS and LABA combinations ordered; total no. of ICS, LABA, and LAMA combinations ordered; total no. of LABA and LAMA combinations ordered; and total no. of SABA and SAMA combinations ordered. | |
| Insurance | Computed based on the end of the index year: whether the patient had any private insurance; whether the patient had any public insurance; and whether the patient was paid by oneself or a charity. | Number of features: 3. |
| Encounter | No. of all types of encounters; no. of major encounters for COPD [28,50]; no. of outpatient visits; no. of outpatient visits with a primary diagnosis of COPD; no. of emergency department (ED) visits; average length of stay of an ED visit; no. of ED visits related to COPD [28]; no. of inpatient stays; total length of inpatient stays; average length of an inpatient stay; no. of inpatient stays related to acute COPD exacerbation or respiratory failure; no. of encounters related to acute COPD exacerbation or respiratory failure [18,60]; no. of outpatient visits to the patient's primary care provider (PCP); no. of admissions to the intensive care unit; admission type of the most emergent encounter (elective, urgent, emergency, or trauma); admission type of the last encounter (elective, urgent, emergency, or trauma); type of the last encounter (ED visit, outpatient visit, or inpatient stay); type of the first encounter (ED visit, outpatient visit, or inpatient stay) related to COPD in the data set; length of stay of the last ED visit; no. of ED visits in the past 6 months; no. of inpatient stays in the past 6 months; and no. of major encounters for COPD in the past 6 months. | A major encounter for COPD was defined as an ED visit having a COPD diagnosis code, an inpatient stay having a COPD diagnosis code, or an outpatient visit having a primary diagnosis of COPD. All else being equal and compared with a patient with only outpatient visits with COPD as a secondary diagnosis, a patient with $\geq$1 major encounter for COPD is more likely to have severe COPD exacerbations in the future.<br>Number of features: 22. |
| Visit status and appointment scheduling | The day of the week when the last ED visit started; the last encounter's discharge disposition location (home, left against medical advice, or other non-home location); no. of times of leaving against medical advice; no. of no shows; no. of cancelled appointments; no. of days since the last inpatient stay; no. of days since the last outpatient visit; no. of days since the last outpatient visit on COPD; no. of days since the last ED visit; no. of days since the last ED visit on COPD [62]; the shortest time between making the request and the actual visit among all occurred encounters; no. of days between making the request and the actual visit of the last encounter; no. of visits having same day appointments; and whether the last inpatient stay came from the ED. | Number of features: 14. |
| Patient's care continuity degree | No. of distinct medication prescribers; no. of distinct COPD medication prescribers; no. of distinct providers seen in outpatient visits; no. of distinct PCPs of the patient; and no. of distinct ED locations the patient went to (including inpatient stays admitted from the ED). | Number of features: 5. |
| Procedure | No. of CPT and HCPCS procedure codes; no. of ICD-10 and ICD-9 procedure codes; no. of CPT procedure codes of the fractional exhaled nitric oxide test; no. of CPT procedure codes of spirometry; no. of HCPCS procedure codes of home oxygen therapy; no. of CPT and HCPCS procedure codes of influenza vaccination; and whether mechanical ventilation was recorded using ICD-10 and ICD-9 procedure codes. | Number of features: 7. |
| Allergy | No. of the patient's allergies; indicator of food allergy; indicator of drug or material allergy; indicator of environmental allergy; the greatest severity of the patient's food allergies; the greatest severity of the | Number of features: 7. |

| | | |
|---|---|---|
| | patient's drug or material allergies; and the greatest severity of the patient's environmental allergies. | |
| Social behavior history | Whether the patient was last recorded as a current smoker; whether the patient was last recorded as a former smoker; the last recorded no. of packs of cigarettes the patient consumed per day; the average no. of packs of cigarettes the patient consumed per day across all of the records; no. of years the patient had smoked for based on the last record; whether the patient was ever documented of consuming alcohol; whether the patient consumed alcohol based on the last record; the last recorded no. of fluid ounces of alcohol the patient consumed per week; the average no. of fluid ounces of alcohol the patient consumed per week across all of the records; the last recorded no. of alcohol drinks the patient consumed per week; the average no. of alcohol drinks the patient consumed per week across all of the records; whether the patient took any illicit drug based on the last record; whether the patient was ever documented of taking any illicit drug; the last recorded no. of times the patient took illicit drugs per week; and the average no. of times the patient took illicit drugs per week across all of the records. | Number of features: 15. |
| Provider | The PCP's type (physician, nurse, physician assistant, or other); whether the PCP is a resident; the PCP's clinician title (Doctor of Medicine, registered nurse, physician assistant, or other); the PCP's age; whether the patient and the PCP are of the same gender; no. of years for which the PCP had practiced at the UWM; no. of patients with COPD of the PCP; and the percentage of the PCP's patients with COPD in the pre-index year having severe COPD exacerbations in the index year. | A patient's current PCP was defined as the patient's PCP known at the last outpatient visit. Number of features: 8. |

**Table S2.** The features employed in the final model in the main analysis and their importance values.

| Rank | Feature | Importance value in % based on the feature's apportioned contribution to the model |
|---|---|---|
| 1 | Number of days since the last diagnosis code of acute COPD exacerbation | 8.384 |
| 2 | Number of COPD diagnoses | 5.287 |
| 3 | Number of diagnoses of acute COPD exacerbation | 5.107 |
| 4 | Number of days since the last ED visit | 4.851 |
| 5 | Number of major encounters | 4.583 |
| 6 | Number of primary or principal COPD diagnoses | 3.571 |
| 7 | Number of days since the last COPD diagnosis code | 2.249 |
| 8 | Average $SpO_2$ | 2.016 |
| 9 | Maximum body mass index | 2.003 |
| 10 | Number of ED visits | 1.947 |
| 11 | Number of ED visits in the past 6 months | 1.900 |
| 12 | Number of years from the first encounter related to COPD | 1.846 |
| 13 | Whether the patient is married | 1.659 |
| 14 | Number of days since the last ED visit related to COPD | 1.534 |
| 15 | Average length of stay of an ED visit | 1.521 |
| 16 | Average respiratory rate | 1.275 |
| 17 | Number of CPT procedure codes | 1.128 |
| 18 | Average heart rate | 1.056 |
| 19 | Total number of distinct medications ordered | 1.013 |
| 20 | Number of no shows | 0.994 |
| 21 | Minimum $SpO_2$ | 0.990 |
| 22 | Average temperature | 0.965 |
| 23 | Whether the first COPD diagnosis was given at an outpatient visit | 0.951 |
| 24 | Number of encounters related to acute COPD exacerbation or respiratory failure | 0.928 |

| | | |
|---|---|---|
| 25 | Number of days between making the request and the actual visit of the last encounter | 0.864 |
| 26 | Whether the patient is Hispanic | 0.834 |
| 27 | Number of days since the last laboratory test | 0.802 |
| 28 | Whether the admission type of the most emergent encounter was elective | 0.758 |
| 29 | Number of medication orders | 0.748 |
| 30 | Total number of distinct medications in COPD reliever orders | 0.708 |
| 31 | Whether the admission type of the most emergent encounter was emergency | 0.703 |
| 32 | Relative change of body mass index | 0.661 |
| 33 | Number of ICD-9 and ICD-10 diagnosis codes | 0.658 |
| 34 | Number of days since the last outpatient visit | 0.656 |
| 35 | Maximum heart rate | 0.649 |
| 36 | Day of the week when the last ED visit started | 0.631 |
| 37 | Breathing abnormality like dyspnea | 0.582 |
| 38 | Relative change of weight | 0.580 |
| 39 | Total number of COPD medication refills allowed | 0.557 |
| 40 | Maximum red blood cell count | 0.548 |
| 41 | Maximum temperature | 0.546 |
| 42 | Maximum height | 0.540 |
| 43 | Maximum Hgb level | 0.537 |
| 44 | Maximum white blood cell count | 0.534 |
| 45 | Total number of units of long-acting muscarinic antagonists ordered | 0.530 |
| 46 | Average diastolic blood pressure | 0.516 |
| 47 | Total number of distinct medications in COPD medication orders | 0.499 |
| 48 | Average systolic blood pressure | 0.493 |
| 49 | Age | 0.468 |
| 50 | Maximum eosinophil count | 0.467 |
| 51 | Total number of units of medications ordered | 0.466 |
| 52 | Average length of an inpatient stay | 0.463 |
| 53 | Total number of medications ordered | 0.460 |
| 54 | The shortest time between making the request and the actual visit among all occurred encounters | 0.457 |
| 55 | Number of days since the last outpatient visit on COPD | 0.454 |
| 56 | Number of COPD medication orders | 0.439 |
| 57 | Number of nebulizer medication orders | 0.434 |
| 58 | Maximum neutrophil count | 0.434 |
| 59 | Whether the patient is a black or an African American | 0.421 |
| 60 | Maximum percentage of eosinophils | 0.419 |
| 61 | Number of major encounters in the past 6 months | 0.414 |
| 62 | Maximum systolic blood pressure | 0.400 |
| 63 | Number of years the patient had smoked for based on the last record | 0.399 |
| 64 | Number of laboratory tests | 0.379 |
| 65 | Maximum percentage of neutrophils | 0.374 |
| 66 | Average number of packs of cigarettes the patient smoked per day across all of the records | 0.368 |
| 67 | Number of ED visits related to COPD | 0.364 |
| 68 | Number of active problems | 0.360 |
| 69 | Length of stay of the last ED visit | 0.354 |
| 70 | Total number of medication refills allowed | 0.354 |
| 71 | Maximum respiratory rate | 0.349 |
| 72 | Maximum diastolic blood pressure | 0.341 |
| 73 | Total number of units of COPD controllers ordered | 0.335 |
| 74 | Minimum Hgb level | 0.332 |
| 75 | Total length of inpatient stays | 0.322 |
| 76 | Number of outpatient visits to the PCP | 0.318 |
| 77 | Maximum $PaO_2$ | 0.312 |

| | | |
|---|---|---|
| 78 | Minimum Hct | 0.301 |
| 79 | Total number of refills allowed for COPD controllers | 0.299 |
| 80 | Total number of medications in nebulizer medication orders | 0.298 |
| 81 | Total number of short-acting beta-2 agonists ordered | 0.296 |
| 82 | Whether the first COPD diagnosis was given at an ED visit | 0.295 |
| 83 | Number of CPT and HCPCS procedure codes of influenza vaccination | 0.291 |
| 84 | Number of laboratory tests with abnormal results | 0.288 |
| 85 | The last recorded number of packs of cigarettes the patient consumed per day | 0.288 |
| 86 | Whether the patient was last recorded as a current smoker | 0.283 |
| 87 | Total number of long-acting muscarinic antagonists ordered | 0.269 |
| 88 | Number of distinct medication prescribers | 0.268 |
| 89 | Total number of long-acting beta-2 agonists ordered | 0.260 |
| 90 | Number of cancelled appointments | 0.259 |
| 91 | Whether the patient is a white | 0.257 |
| 92 | Maximum Hct | 0.249 |
| 93 | Total number of units of COPD medications ordered | 0.223 |
| 94 | Maximum HbA1c | 0.223 |
| 95 | Total number of refills allowed for long-acting muscarinic antagonists | 0.223 |
| 96 | Total number of COPD medications ordered | 0.222 |
| 97 | Total number of refills allowed for inhaled corticosteroids | 0.219 |
| 98 | Number of same day appointments | 0.213 |
| 99 | Priority of the last active problem of COPD | 0.212 |
| 100 | Number of all types of encounters | 0.211 |
| 101 | Total number of units of COPD relievers ordered | 0.211 |
| 102 | Total number of refills allowed for long-acting beta-2 agonists | 0.209 |
| 103 | Whether the patient's PCP is a Doctor of Medicine | 0.208 |
| 104 | Total number of inhaled corticosteroid and long-acting beta-2 agonist combinations ordered | 0.206 |
| 105 | Number of COPD controller orders | 0.195 |
| 106 | Number of COPD reliever orders | 0.194 |
| 107 | Total number of distinct medications in nebulizer medication orders | 0.187 |
| 108 | Number of distinct ED locations the patient went to (including inpatient stays admitted from the ED) | 0.187 |
| 109 | Maximum CRP | 0.185 |
| 110 | Whether the last encounter was an ED visit | 0.185 |
| 111 | Total number of inhaled corticosteroids ordered | 0.184 |
| 112 | Number of ICD-10 and ICD-9 procedure codes | 0.172 |
| 113 | Total number of units of short-acting muscarinic antagonists ordered | 0.172 |
| 114 | Total number of units of nebulizer medications ordered | 0.172 |
| 115 | Minimum $PaCO_2$ | 0.172 |
| 116 | Whether the last encounter's discharge disposition location was home | 0.170 |
| 117 | Total number of refills allowed for short-acting beta-2 agonists | 0.169 |
| 118 | Total number of short-acting muscarinic antagonists ordered | 0.165 |
| 119 | Number of active problems about smoking | 0.163 |
| 120 | Number of days since the last inpatient stay | 0.162 |
| 121 | Number of distinct COPD medication prescribers | 0.161 |
| 122 | Total number of distinct medications in COPD controller orders | 0.148 |
| 123 | The greatest severity of the patient's drug or material allergies | 0.143 |
| 124 | Total number of units of inhaled corticosteroids ordered | 0.143 |
| 125 | Number of active problems of COPD | 0.140 |
| 126 | Total number of short-acting beta-2 agonist and short-acting muscarinic antagonist combinations ordered | 0.137 |
| 127 | Total number of units of short-acting beta-2 agonists ordered | 0.132 |
| 128 | Total number of systemic corticosteroids ordered | 0.128 |

| | | |
|---|---|---|
| 129 | Number of outpatient visits | 0.126 |
| 130 | Whether the last COPD diagnosis was a primary or principal diagnosis | 0.121 |
| 131 | Number of patients with COPD of the PCP | 0.120 |
| 132 | Average number of fluid ounces of alcohol the patient consumed per week across all of the records | 0.116 |
| 133 | Number of allergies | 0.116 |
| 134 | Age of the PCP | 0.113 |
| 135 | Total number of medications in COPD controller orders | 0.109 |
| 136 | Total number of medications in COPD reliever orders | 0.109 |
| 137 | The last recorded number of alcohol drinks the patient consumed per week | 0.108 |
| 138 | Whether the patient was paid by oneself or a charity on the last day | 0.107 |
| 139 | The last recorded number of fluid ounces of alcohol the patient consumed per week | 0.106 |
| 140 | Whether the patient is divorced | 0.095 |
| 141 | Whether the patient was last recorded as a former smoker | 0.093 |
| 142 | Whether the patient is widowed | 0.093 |
| 143 | Average number of alcohol drinks the patient consumed per week across all of the records | 0.092 |
| 144 | Minimum $SaO_2$ | 0.087 |
| 145 | The percentage of the PCP's patients with COPD in the pre-index year having severe COPD exacerbations in the index year | 0.087 |
| 146 | Whether the patient's PCP is a physician | 0.087 |
| 147 | Whether the patient had any private insurance on the last day | 0.085 |
| 148 | Number of CPT procedure codes of spirometry | 0.084 |
| 149 | Total number of refills allowed for COPD relievers | 0.082 |
| 150 | Total number of units of long-acting beta-2 agonists ordered | 0.080 |
| 151 | Number of active problems of hypertension | 0.079 |
| 152 | Total number of refills allowed for short-acting muscarinic antagonists | 0.078 |
| 153 | Whether the patient took any illicit drug based on the last record | 0.078 |
| 154 | Number of inpatient stays related to acute COPD exacerbation or respiratory failure | 0.076 |
| 155 | Whether the patient is single | 0.074 |
| 156 | Minimum $PaO_2$ | 0.074 |
| 157 | Number of active problems of sleep apnea | 0.073 |
| 158 | Whether the patient had any public insurance on the last day | 0.072 |
| 159 | Peripheral vascular disease | 0.071 |
| 160 | Number of distinct providers seen in outpatient visits | 0.070 |
| 161 | Upper respiratory tract infection | 0.068 |
| 162 | Substance use | 0.065 |
| 163 | Whether a nebulizer was used | 0.065 |
| 164 | Whether the patient has any material or drug allergy | 0.061 |
| 165 | Whether the patient used a spacer | 0.059 |
| 166 | Number of distinct PCPs of the patient | 0.058 |
| 167 | Maximum $PaCO_2$ | 0.057 |
| 168 | Number of outpatient visits with a primary diagnosis of COPD | 0.057 |
| 169 | Total number of refills allowed for nebulizer medications | 0.057 |
| 170 | Number of inpatient stays | 0.054 |
| 171 | Congestive heart failure | 0.054 |
| 172 | Whether the patient consumed alcohol based on the last record | 0.054 |
| 173 | Dementia | 0.051 |
| 174 | Whether the patient is an Asian | 0.049 |
| 175 | Total number of units of systemic corticosteroids ordered | 0.048 |
| 176 | Mental disorder | 0.046 |
| 177 | Number of inpatient stays in the past 6 months | 0.045 |
| 178 | Whether the patient was ever documented of consuming alcohol | 0.043 |
| 179 | Cerebrovascular disease | 0.041 |
| 180 | Whether the patient was ever documented of taking any illicit drug | 0.039 |

| | | |
|---|---|---|
| 181 | Metastatic solid tumor | 0.038 |
| 182 | Whether the last inpatient stay came from the ED | 0.038 |
| 183 | Number of active problems of diabetes | 0.036 |
| 184 | Number of active problems of anxiety/depression | 0.034 |
| 185 | Hypertension | 0.033 |
| 186 | Renal disease | 0.033 |
| 187 | Number of active problems of acute COPD exacerbation | 0.030 |
| 188 | Obesity | 0.029 |
| 189 | Whether the maximum Hgb was abnormally high | 0.027 |
| 190 | Number of active problems of gastroesophageal reflux disease | 0.027 |
| 191 | Gastroesophageal reflux | 0.027 |
| 192 | Asthma | 0.026 |
| 193 | Whether the patient speaks English | 0.025 |
| 194 | Myocardial infarction | 0.023 |
| 195 | Total number of refills allowed for systemic corticosteroids | 0.022 |
| 196 | Whether the patient is a female | 0.020 |
| 197 | Whether the patient is separated from the spouse | 0.020 |
| 198 | Diabetes without chronic complication | 0.020 |
| 199 | Whether the last encounter was an inpatient stay | 0.020 |
| 200 | Whether the minimum Hct was abnormally low | 0.019 |
| 201 | Whether the admission type of the last encounter was elective | 0.018 |
| 202 | Whether the maximum Hct was abnormally high | 0.017 |
| 203 | Average peak expiratory flow rate | 0.016 |
| 204 | Whether the patient had undergone mechanical ventilation | 0.016 |
| 205 | Lung cancer | 0.016 |
| 206 | Whether the patient is a Native Hawaiian or an other Pacific Islander | 0.015 |
| 207 | Mild liver disease | 0.014 |
| 208 | Cirrhosis | 0.014 |
| 209 | Hemiplegia or paraplegia | 0.014 |
| 210 | Number of diagnoses of noncompliance with medication regimen | 0.013 |
| 211 | Pneumonia | 0.013 |
| 212 | Whether the patient's PCP's title is nurse | 0.013 |
| 213 | Ischemic heart disease | 0.013 |
| 214 | Acquired immunodeficiency syndrome | 0.013 |
| 215 | Whether the patient has any environmental allergy | 0.011 |
| 216 | Vitamin D deficiency | 0.011 |
| 217 | Number of active problems of congestive heart failure | 0.010 |
| 218 | The last recorded number of times the patient took illicit drugs per week | 0.010 |
| 219 | Malignancy | 0.008 |
| 220 | Number of active problems of dyspnea | 0.008 |
| 221 | Number of active problems of asthma | 0.008 |
| 222 | Gastrointestinal bleeding | 0.008 |
| 223 | Average number of times the patient took illicit drugs per week across all of the records | 0.008 |
| 224 | Cataract | 0.007 |
| 225 | Diabetes with chronic complication | 0.006 |
| 226 | Number of active problems of rhinitis | 0.006 |
| 227 | Whether the minimum Hgb was abnormally low | 0.006 |
| 228 | Number of active problems of obesity | 0.005 |
| 229 | Allergic rhinitis | 0.004 |

We used the xgb.save() function in the xgboost package of R to save our final XGBoost model to a file in binary format. This file is available at http://faculty.washington.edu/luogang/COPD_care_model_UW.

**Abbreviations**:

A1AT: Alpha-1 antitrypsin
BMI: body mass index
COPD: chronic obstructive pulmonary disease
CPT: Current Procedural Terminology
CRP: C-reactive protein
ED: emergency department
$FEV_1$: forced expiratory volume in 1 second
FVC: forced vital capacity
HCPCS: Healthcare Common Procedure Coding System
Hct: hematocrit
HbA1c: hemoglobin A1c
Hgb: hemoglobin
ICD-10: International Classification of Diseases, Tenth Revision
ICD-9: International Classification of Diseases, Ninth Revision
ICS: inhaled corticosteroid
IgA: immunoglobulin A
IgE: immunoglobulin E
LABA: long-acting beta-2 agonist
LAMA: long-acting muscarinic antagonist
$PaCO_2$: arterial partial pressure of carbon dioxide
$PaO_2$: arterial partial pressure of oxygen
PCP: primary care provider
PDE-4: phosphodiesterase-4 inhibitor
SABA: short-acting beta-2 agonist
SAMA: short-acting muscarinic antagonist
$SaO_2$: arterial oxygen saturation
$SpO_2$: peripheral capillary oxygen saturation

**References**


3. Global Initiative for Chronic Obstructive Lung Disease - GOLD. 2020 Gold Reports. 2020. https://goldcopd.org/gold-reports.
18. Hurst JR, Vestbo J, Anzueto A, Locantore N, Müllerova H, Tal-Singer R, Miller B, Lomas DA, Agusti A, Macnee W, Calverley P, Rennard S, Wouters EF, Wedzicha JA; Evaluation of COPD Longitudinally to Identify Predictive Surrogate Endpoints (ECLIPSE) Investigators. Susceptibility to exacerbation in chronic obstructive pulmonary disease. N Engl J Med 2010 Sep 16;363(12):1128-1138. PMID:20843247
28. Alcázar B, García-Polo C, Herrejón A, Ruiz LA, de Miguel J, Ros JA, García-Sidro P, Conde GT, López-Campos JL, Martínez C, Costán J, Bonnin M, Mayoralas S, Miravitlles M. Factors associated with hospital admission for exacerbation of chronic obstructive pulmonary disease. Arch Bronconeumol 2012 Mar;48(3):70-76. PMID:22196478
30. Miravitlles M, Guerrero T, Mayordomo C, Sánchez-Agudo L, Nicolau F, Segú JL. Factors associated with increased risk of exacerbation and hospital admission in a cohort of ambulatory COPD patients: a multiple logistic regression analysis. The EOLO Study Group. Respiration 2000;67(5):495-501. PMID:11070451
50. Niewoehner DE, Lokhnygina Y, Rice K, Kuschner WG, Sharafkhaneh A, Sarosi GA, Krumpe P, Pieper K, Kesten S. Risk indexes for exacerbations and hospitalizations due to COPD. Chest 2007 Jan;131(1):20-28. PMID:17218552
59. Qureshi H, Sharafkhaneh A, Hanania NA. Chronic obstructive pulmonary disease exacerbations: latest evidence and clinical implications. Ther Adv Chronic Dis 2014 Sep;5(5):212-227. PMID:25177479
60. Müllerova H, Maselli DJ, Locantore N, Vestbo J, Hurst JR, Wedzicha JA, Bakke P, Agusti A, Anzueto A. Hospitalized exacerbations of COPD: risk factors and outcomes in the ECLIPSE cohort. Chest 2015 Apr;147(4):999-1007. PMID:25356881
61. Donaldson GC, Seemungal TA, Bhowmik A, Wedzicha JA. Relationship between exacerbation frequency and lung function decline in chronic obstructive pulmonary disease. Thorax 2002 Oct;57(10):847-852. PMID:12324669
62. Hurst JR, Donaldson GC, Quint JK, Goldring JJ, Baghai-Ravary R, Wedzicha JA. Temporal clustering of exacerbations in chronic obstructive pulmonary disease. Am J Respir Crit Care Med 2009 Mar 1;179(5):369-374. PMID:19074596
63. Similowski T, Agustí A, MacNee W, Schönhofer B. The potential impact of anaemia of chronic disease in COPD. Eur Respir J 2006 Feb;27(2):390-396. PMID:16452598

64. Dahl M, Vestbo J, Lange P, Bojesen SE, Tybjaerg-Hansen A, Nordestgaard BG. C-reactive protein as a predictor of prognosis in chronic obstructive pulmonary disease. Am J Respir Crit Care Med 2007 Feb 1;175(3):250-255. PMID:17053205
65. Hoenderdos K, Condliffe A. The neutrophil in chronic obstructive pulmonary disease. Am J Respir Cell Mol Biol 2013 May;48(5):531-539. PMID:23328639
66. Lonergan M, Dicker AJ, Crichton ML, Keir HR, Van Dyke MK, Mullerova H, Miller BE, Tal-Singer R, Chalmers JD. Blood neutrophil counts are associated with exacerbation frequency and mortality in COPD. Respir Res 2020 Jul 1;21(1):166. PMID:32611352
67. Chambellan A, Chailleux E, Similowski T; ANTADIR Observatory Group. Prognostic value of the hematocrit in patients with severe COPD receiving long-term oxygen therapy. Chest 2005 Sep;128(3):1201-1208. PMID:16162707
68. Toft-Petersen AP, Torp-Pedersen C, Weinreich UM, Rasmussen BS. Association between hemoglobin and prognosis in patients admitted to hospital for COPD. Int J Chron Obstruct Pulmon Dis 2016 Nov 10;11:2813-2820. PMID:27877035
69. van Dijk EJ, Vermeer SE, de Groot JC, van de Minkelis J, Prins ND, Oudkerk M, Hofman A, Koudstaal PJ, Breteler MM. Arterial oxygen saturation, COPD, and cerebral small vessel disease. J Neurol Neurosurg Psychiatry 2004 May;75(5):733-736. PMID:15090569
70. Kessler R, Faller M, Fourgaut G, Mennecier B, Weitzenblum E. Predictive factors of hospitalization for acute exacerbation in a series of 64 patients with chronic obstructive pulmonary disease. Am J Respir Crit Care Med 1999 Jan;159(1):158-164. PMID:9872834
71. Fermont JM, Masconi KL, Jensen MT, Ferrari R, Di Lorenzo VAP, Marott JM, Schuetz P, Watz H, Waschki B, Müllerova H, Polkey MI, Wilkinson IB, Wood AM. Biomarkers and clinical outcomes in COPD: a systematic review and meta-analysis. Thorax 2019 May;74(5):439-446. PMID:30617161
72. Halpin DM, Miravitlles M, Metzdorf N, Celli B. Impact and prevention of severe exacerbations of COPD: a review of the evidence. Int J Chron Obstruct Pulmon Dis 2017 Oct 5;12:2891-2908. PMID:29062228
102. Guinness World Records. The world’s oldest people and their secrets to a long life. 2020. https://www.guinnessworldrecords.com/news/2020/10/the-worlds-oldest-people-and-their-secrets-to-a-long-life-632895.
103. Guinness World Records. Lightest birth. 2020. https://www.guinnessworldrecords.com/world-records/lightest-birth.
104. Guinness World Records. Heaviest man ever. 2020. https://www.guinnessworldrecords.com/world-records/heaviest-man.
105. Guinness World Records. Shortest baby. 2020. https://www.guinnessworldrecords.com/world-records/shortest-baby.
106. Guinness World Records. Tallest man ever. 2020. https://www.guinnessworldrecords.com/world-records/tallest-man-ever.
107. Gwyneth O. Part V Fat: no more fear, no more contempt. The Eating Disorder Institute. 2011. https://edinstitute.org/blog/2011/12/8/part-v-fat-no-more-fear-no-more-contempt.
108. Wikipedia. List of heaviest people. 2021. https://en.wikipedia.org/w/index.php?title=List_of_heaviest_people&oldid=1000662342.
109. Hankinson JL, Odencrantz JR, Fedan KB. Spirometric reference values from a sample of the general U.S. population. Am J Respir Crit Care Med 1999 Jan;159(1):179-187. PMID:9872837
110. Pellegrino R, Viegi G, Brusasco V, Crapo RO, Burgos F, Casaburi R, Coates A, van der Grinten CP, Gustafsson P, Hankinson J, Jensen R, Johnson DC, MacIntyre N, McKay R, Miller MR, Navajas D, Pedersen OF, Wanger J. Interpretative strategies for lung function tests. Eur Respir J 2005 Nov;26(5):948-968. PMID:16264058
111. Marion MS, Leonardson GR, Rhoades ER, Welty TK, Enright PL. Spirometry reference values for American Indian adults: results from the Strong Heart Study. Chest 2001 Aug;120(2):489-495. PMID:11502648
112. National Jewish Health. Bronchodilators. 2018. https://nationaljewish.org/conditions/medications/copd/bronchodilators.
